# The Mediating Role of Emotion Regulation Difficulties in the Relationship Between Experiential Avoidance and Somatic Symptoms: A Cross-Sectional Study

**DOI:** 10.3390/bs16050795

**Published:** 2026-05-16

**Authors:** Erinç Erbildim, Gabriel Elochukwu Nweke

**Affiliations:** 1 Faculty of Humanities and Social Sciencs, Cyprus Aydın University, Girne 99320, Cyprus; 2 Faculty of Humanities, Girne American University, Girne 99138, Cyprus; nwekegabriel@gau.edu.tr

**Keywords:** somatic symptoms, experiential avoidance, difficulties in emotion regulation

## Abstract

Experiential avoidance, defined as unwillingness to deal with personal experiences such as thoughts, emotions, and memories, is closely related to difficulties in emotion regulation. This is because emotional awareness and acceptance are crucial for regulating distressing feelings. Somatic symptoms, referring to bodily sensations such as headaches, nausea, and fatigue with or without any underlying medical condition, are frequently reported among individuals with avoided or dysregulated emotional burden. This cross-sectional correlational study aimed to examine the mediating role of emotion regulation difficulties in the relationship between experiential avoidance and somatic symptoms; we used a sample size of 397 individuals recruited from a non-clinical population with the convenience sampling technique. The measurement instruments were the Brief Experiential Avoidance Questionnaire (BEAQ), Somatic Symptom Scale (SSS-8), and Difficulties in Emotion Regulation Scale (DERS-16). Statistical analysis was conducted using the IBM SPSS 29 statistical program and the SPSS Process Macro 4.2 extension. The results indicate that difficulties in emotion regulation mediated the relationship between experiential avoidance and somatic symptoms controlling for age, education, gender and perceived income and all variables were significantly correlated with each other, including subscales of difficulties in emotion regulation. Limited access to emotion regulation strategies was subscale with an indirect effect on the association between experiential avoidance and somatic symptoms. These findings are expected to guide mental health professionals in consulting clients with somatic symptoms and emotion regulation difficulties.

## 1. Introduction

Experiential avoidance refers to the unwillingness to deal with internal experiences, such as thoughts, emotions, mental images, and memories. It involves taking actions intended to reduce the intensity of these experiences or modify them. In the long term, avoidance is dysfunctional as a coping mechanism and causes further psychological distress despite providing short-term relief (S. C. Hayes et al., 2004). Consistent avoidance of internal experiences is associated with detachment from reality and the present moment, interfering with daily functioning (Kashdan et al., 2006).

Two forms of experiential avoidance are suppression and situational avoidance. Suppression involves attempts to control and reduce the intensity of immediate internal experiences such as distressing thoughts, emotions, memories, or physical symptoms. Situational avoidance aims to modify external factors acting as triggers of these experiences (S. C. Hayes et al., 1999). According to S. C. Hayes et al. (1996), all attempts and methods to modify personal experiences by escaping and avoiding are a part of the concept of experiential avoidance. Individuals who engage in experiential avoidance expect to regulate distressing emotions and feel protected from the possible catastrophic consequences of these experiences. Despite serving as an effective short-term emotion regulation approach, avoidance requires a significant amount of time and energy, impairing one’s progress towards achieving their life goals and limiting their availability of emotional experiences (Kashdan et al., 2006).

Experiential avoidance is associated with psychopathological symptoms and disorders. Kato (2024) found that attempts to avoid internal experiences are related to excessive rumination, which means repetitive thinking of a past event without engaging in active problem solving or coping. Suppressing thoughts to avoid distressing cognitions is common among individuals with borderline personality disorder, which is characterized by unstable mood and impaired self-image and interpersonal relationships. Chapman et al. (2005) further concluded that experiential avoidance and self-harm tendencies are also positively correlated (Chapman et al., 2005). Experiential avoidance mediates past family-conflict-related emotions and depressive symptoms according to Biglan et al. (2015). Their study shows that avoiding emotional burden from the past might contribute to the development of depressive symptoms during adulthood. Distortion in the capacity of working memory and cognitive capacities such as judgment and reasoning is observed among individuals who engage in experiential avoidance in the long term. These skills play a crucial role in the process of emotion regulation. Thus, experiential avoidance is expected to contribute to emotion regulation difficulties, thus impairing cognitive skills (Karademas et al., 2017). Experiential avoidance is also observed to be significantly higher among individuals with anxiety disorders (Berghoff et al., 2017). Additionally, how one responds to an internal or external stimulus (avoidant or accepting) is a determinant of further physical symptoms in the presence of an anxiety disorder.

Somatic symptoms refer to physical symptoms with or without any organic basis (Fink & Rosendal, 2008). Somatic symptoms are real and can be perceived like other symptoms without being attributed to a medical condition. For instance, whilst some types of headaches are associated with a condition such as a brain tumor or migraine, some types of headaches occur without any underlying diagnosis and become more intense during times of perceived stress or when experiencing a psychological condition. Other common somatic symptoms are abdominal pain, nausea, fatigue, dizziness, and heart palpitations (Eriksen & Ursin, 2004).

Somatic experiences mostly include a group of symptoms rather than a single symptom within the body. This group of physical manifestations is called functional somatic syndromes, characterized by distress and impairment in daily functioning (Barsky & Borus, 1999). Fibromyalgia, chronic fatigue syndrome, and irritable bowel syndrome are examples of functional somatic syndromes that have an unclear physical diagnosis and are attributed to psychological factors (Burton, 2003).

The psychodynamic model offers one of the first explanations for somatic symptoms, implying that they reflect psychological conflicts from childhood (Kirmayer & Young, 1998). It is stated that anxiety-related impulses are channeled into physical symptoms and complaints through the autonomic nervous system to repress anxiety from consciousness. Long-term repression of emotional and cognitive experiences disturbs the physiology of the physical body, causing persistent somatic symptoms (Menniger, 1947). Numbness is associated with conflicts in the past that are avoided or not resolved, and pain in the pelvic area is attributed to childhood sexual abuse (Simon et al., 1996). According to Kirmayer and Young (1998), psychic energy avoided with defense mechanisms will eventually manifest as physical symptoms, leaving one without conscious awareness of their psychological distress. Individuals with these persistent bodily symptoms might attribute them to a medical condition and seek physical treatment. In the psychodynamic model, these individuals are described as psychologically defended as they avoid emotional expression and conflicts.

Childhood maltreatment, such as emotional abuse or neglect, is associated with somatization during adulthood. Maltreated children might feel overwhelmed by abuse-related emotions and suppress them, avoiding emotional awareness to feel protected in the presence of difficult emotions and thoughts (Fonagy & Allison, 2012). In addition, children growing up under non-nurturing circumstances where emotional expression is punished or neglected might lack the emotion regulation skills needed to cope with emotional experiences, which manifest as somatic symptoms. Zulkifli et al. (2025) indicated that somatic symptom intensity is positively associated with cognitive and emotional avoidance.

Emotion regulation involves internal and external processes to observe, evaluate, and adjust the intensity of emotional reactions (Thompson, 1990). While emotions have crucial roles in providing guidance for external and internal experiences and motivation, a lack of emotional awareness and regulation skills impairs functioning, as distressing emotions may interfere with goal-directed behavior (Cicchetti & Toth, 1995). Gross (1998) defines emotion regulation as expression and modulation of emotions. Various activities can be considered emotion regulatory, such as punching a pillow when feeling angry, chatting with friends when feeling sad, or planning a fun activity to elicit positive emotions.

According to the hedonic approach, emotion regulation aims to down-regulate negative emotions such as anger and sadness by adjusting intensity and duration or up-regulating positive emotions such as joy and motivation (Gross et al., 2006). Larsen’s (2000) finding is consistent with this implication indicating that individuals are motivated to reduce the intensity and duration of negative affect and increase positive affect. The aim of emotion regulation is not only to modulate emotions but also to modify behavior and motivation. Parrott (1993) mentions that people are sometimes motivated to upregulate negative emotions to provide a focused mindset, engage in empathic perspectives, and have influence on others. Additionally, down-regulating positive emotions helps one to have a more realistic perspective of the self and the world and become aware of suppressed feelings. This broader framework indicates that individuals are sometimes motivated to regulate emotions for short-term pleasure and pain avoidance, and at other times, emotion regulation helps to clarify one’s mindset and increase awareness (Mauss & Tamir, 2014).

Difficulties in emotion regulation involve patterns that interfere with one’s goal-oriented motivation and behavior (Cole et al., 2017). Maladaptive emotion regulation strategies or a lack of effective regulation skills are associated with difficulties in emotion regulation. According to Cicchetti and Toth (1995), maltreatment during childhood might prevent an individual from adopting adaptive emotion regulation strategies through modeling. In addition, punishment of emotional expressions is associated with a suppressive emotion regulation approach rather than expression and awareness (Kim & Cicchetti, 2010). Family environments perceived as a threat increase the chances of an individual engaging in maladaptive, avoidant emotion regulation approaches with a self-preserving attitude (Frankenhuis & Del Giudice, 2012).

Difficulties in emotion regulation are associated with psychological disorders and overall life satisfaction. Anxiety disorders and internet addiction are observed to be more common among individuals who struggle with regulating their emotions (Akdeniz & Birekul, 2024). According to Saxena et al. (2011), emotion regulation difficulties such as struggling with identifying emotions, lacking emotional clarity, and limited access to emotion regulation strategies are related to decreased life satisfaction and psychological well-being. Emotion regulation plays a critical role in how an individual perceives themself, and those who lack relevant skills tend to be more prone to self-criticism (Rostami et al., 2023). Somatic symptoms are also considered to stem from emotion regulation difficulties. Erbildim and Nweke (2025) revealed that emotion regulation difficulties have a significant indirect association with shame-proneness and somatic symptoms. Furthermore, parents of children with somatic burden were found to struggle with emotion regulation processes and have alexithymic traits (Fostini et al., 2025). These studies demonstrate how somatic symptoms are related to emotional burden that is left unregulated. Trincas et al. (2016) demonstrate that experiential avoidance and difficulties in emotion regulation are closely related. Their study explains that individuals holding negative beliefs about emotions engage in experiential avoidance more often, contributing to emotion regulation difficulties such as having less clarity about their feelings.

Overall, both experiential avoidance and difficulties in emotion regulation have possible predictor roles in the development of somatic symptoms, as avoided and dysregulated emotions are associated with bodily experiences. Experiential avoidance is negatively associated with difficulties in emotion regulation as emotional coping requires acceptance of internal experiences for emotional clarity, appropriate choice of regulation strategy and motivation for change. Therefore, experiential avoidance is conceptualized as a predictor of emotion dysregulation. This study aims to investigate potential mediating role of difficulties in emotion regulation in the relationship between experiential avoidance and somatic symptoms. Additionally, it will be examined whether specific emotion regulation difficulties are associated with the relationship between perceived stress and somatic symptoms via indirect effects, providing insights into potential associations among these variables, thereby highlighting the contribution of individual emotion regulation subscales beyond overall emotion dysregulation.

The hypotheses of the study are as follows:

**H1.**  *There is an association between experiential avoidance and somatic symptoms.*

**H2.**  *There is an association between difficulties in emotion regulation and somatic symptoms.*

**H3.**  *There is an association between experiential avoidance and difficulties in emotion regulation.*

**H4.**  *Difficulties in emotion regulation have a mediating role in the relationship between experiential avoidance and somatic symptoms.*

## 2. Materials and Methods

### 2.1. Participants

A total of 397 adult participants were recruited using a convenience sampling method. Using social media platforms and communication channels, the participants were accessed upon their convenience for participation to complete surveys involving scales for each study variable and demographic questions. The sample size was determined according to power analysis recommendations for mediation analysis (Fritz & MacKinnon, 2007) to ensure adequate statistical power to detect medium-sized effects at a level of 0.05. The inclusion criterion was being at least 18 years of age, with participants representing a general, non-clinical population.

The demographic distribution of the participants is as follows: 48.1% were females (n = 191) and 51.9% were males (n = 206). Regarding educational level, 31% declared that they completed high school, 48.4% held a bachelor’s degree, 17.1% had a master’s degree, and 3.5% held a doctoral degree. Most participants (83.4%) reported having a job, while 16.6% were not working. Regarding perceived income, 33.8% of participants reported their income level as low, 58.9% as average, and 7.3% as high. Marital status distribution was as follows: 45.6% were single, 41.8% were married, 9.6% were divorced, and 3.0% were widowed.

### 2.2. Instruments

Surveys including the sociodemographic questionnaire, Experiential Avoidance Questionnaire (BEAQ), Somatic Symptom Scale (SSS-8), and Difficulties in Emotion Regulation Scale (DERS) were used to collect data regarding sociodemographic status and experiences of the study variables among the participants.

#### 2.2.1. Sociodemographic Questionnaire

The sociodemographic questionnaire was prepared to collect data regarding the participants’ sociodemographic status, such as age, marital status, perceived income, and level of education.

#### 2.2.2. Brief Experiential Avoidance Questionnaire (BEAQ)

The BEAQ is a 15-item questionnaire used to measure the extent of avoidance of internal experiences. The items are scored on a 6-point Likert scale, with total scores ranging between 15 and 90. Higher scores indicate greater avoidance. Cronbach’s alpha values range between 0.80 and 0.89 in previous studies (Gámez et al., 2014), while it is calculated as 0.80 in the current study.

#### 2.2.3. Somatic Symptom Scale (SSS-8)

The SSS-8 is an 8-item scale used to measure somatic burden. The items are scored on a 5-point Likert scale, ranging from 0 (“not at all”) to 4 (“very much”) with higher scores indicating higher levels of somatic burden. Cronbach’s alpha of the scale is 0.81, indicating adequate reliability (Gierk et al., 2014). In the current study, it was calculated as 0.834.

#### 2.2.4. Difficulties in Emotion Regulation Scale (DERS-16)

The DERS-16 is a 16-item inventory with five subscales: nonacceptance of negative emotions, inability to engage in goal-directed behaviors when distressed, difficulties controlling impulsive behaviors when distressed, limited access to emotion regulation strategies perceived as effective, and lack of emotional clarity. Each item is scored on a 5-point Likert scale, ranging from 1 (“almost never”) to 5 (“almost always”), with higher scores indicating greater difficulties in emotion regulation. The Cronbach’s alpha coefficient ranged from 0.92 to 0.95 (Bjureberg et al., 2016); it is 0.726 in the current study.

### 2.3. Procedure

Following ethical approval from the Girne American University Social Sciences Ethics Committee (2024-25/002), data were collected through social media platforms and applications such as Facebook, Instagram, and WhatsApp, as well as by accessing participants in online community groups and community networks who were interested in the study.

The participants were informed about the nature of the study, including the aim of the research, the right to withdraw, the estimated duration for answering the questions (15–20 min), and the basis of voluntariness, with the informed consent form provided prior to the questions to be answered. No identifiable data were collected during the study.

### 2.4. Data Analysis

Statistical analysis was conducted with the SPSS IBM 29 statistical program and the SPSS Process Macro 4.2 extension. Normality assumption was assessed by calculating the skewness and kurtosis of each variable. Values within the range of ±2 for skewness and ±7 for kurtosis were considered normally distributed (West et al., 1995). Correlation analysis was used to examine associations between variables, and mediation analysis was conducted with the PROCESS Macro 4.2 extension (Model 4 and Model 4 with multiple parallel mediators) to estimate the mediating role of difficulties in emotion regulation in the relationship between experiential avoidance and somatic symptoms. Age, gender, income, and education were included as covariates in the mediation models to statistically control for their potential confounding effects. The indirect effects were tested using the bootstrapping method with 5000 resamples to produce bias-corrected 95% confidence intervals (CIs). Bootstrapping is a nonparametric resampling procedure that does not assume normality of the sampling distribution of the indirect effects, making it more robust and reliable, particularly in psychological research with moderate sample sizes (A. F. Hayes, 2018).

## 3. Results

### 3.1. Descriptive Data

Table 1 presents descriptive statistics for the study variables. The mean score for somatic symptoms was 8.31 (SD = 6.44), while experiential avoidance and overall difficulties in emotion regulation had means of 44.71 (SD = 12.97) and 36.02 (SD = 15.26), respectively. Among the subscales of emotion regulation difficulties, inability to engage in goal-directed behavior (M = 8.60, SD = 3.63) had the highest average score, while lack of emotional clarity was the lowest (M = 3.40, SD = 1.87). The skewness and kurtosis values for each variable were within an acceptable range (±2 for skewness and ±7 for kurtosis), supporting the normality assumption. Participants’ ages ranged between 23 and 80, with a mean value of 44.47 and an SD of 12.93.

### 3.2. Correlation Analysis of Variables

Table 2 illustrates the Pearson correlation coefficients among the study variables. Pearson correlation analyses indicated that somatic symptoms were positively and significantly associated with experiential avoidance (r = 0.42, *p* < 0.01) and difficulties in emotion regulation (r = 0.58, *p* < 0.01). Experiential avoidance was also related to difficulties in emotion regulation (r = 0.66, *p* < 0.01). In addition, all subdimensions of emotion regulation difficulties were significantly related to somatic symptoms, with the most significant correlations observed for limited access to emotion regulation strategies (r = 0.56, *p* < 0.01), inability to engage in goal-directed behavior (r = 0.52, *p* < 0.01), and nonacceptance of negative emotions (r = 0.50, *p* < 0.01).

The results show that Hypotheses 1, 2, and 3 are supported as three variables are related.

### 3.3. Mediation Analysis

**Figure 1:**
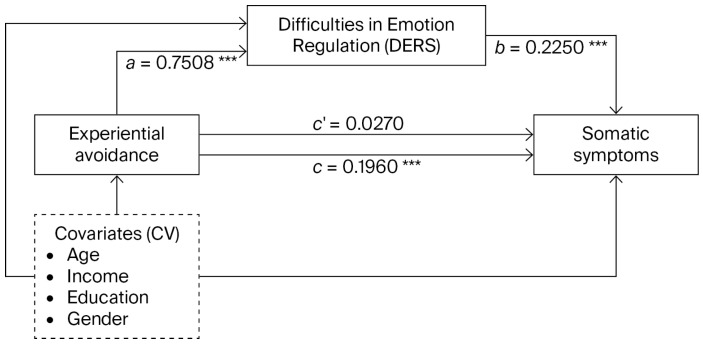
Mediation model examining the association between experiential avoidance and somatic symptoms through difficulties in emotion regulation, controlling for age, income, education, and gender. *** *p* < 0.001.

Figure 1 presents a mediation model that examines whether difficulties in emotion regulation mediate the association between experiential avoidance and somatic symptoms, controlling for age, income, education, and gender. All reported coefficients are unstandardized (b). Experiential avoidance was significantly associated with difficulties in emotion regulation (b = 0.7508, SE = 0.0449, t = 16.73, *p* < 0.001, 95% CI [0.6626, 0.8390]), and difficulties in emotion regulation were significantly associated with somatic symptoms (b = 0.2250, SE = 0.0228, t = 9.87, *p* < 0.001, 95% CI [0.1802, 0.2698]). The direct association between experiential avoidance and somatic symptoms was not statistically significant when controlling for the mediator (b = 0.0270, SE = 0.0265, t = 1.02, *p* = 0.310, 95% CI [−0.0251, 0.0790])**.** The indirect effect of experiential avoidance on somatic symptoms through difficulties in emotion regulation was statistically significant based on 5000 bootstrap samples (b = 0.1690, BootSE = 0.0182, 95% CI [0.1332, 0.2057]), indicating an indirect-only (full) mediation pattern.

Among the covariates, age (β = −0.1118, *p* = 0.013) and income (β = −3.0629, *p* = 0.004) were significantly associated with difficulties in emotion regulation, whereas education (β = −0.9683, *p* = 0.007) and gender (β = −1.5620, *p* = 0.003) were significantly associated with somatic symptoms.

**Figure 2:**
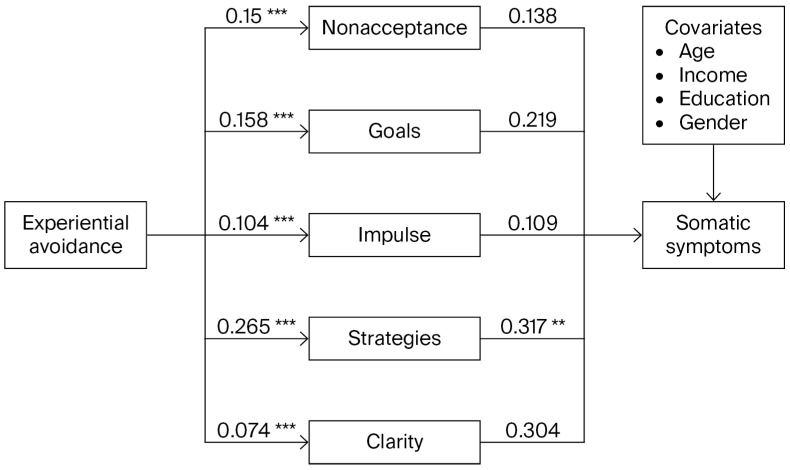
Parallel mediation analysis of difficulties in emotion regulation subscales in the relationship between experiential avoidance and somatic symptoms, controlling for age, income, education, and gender. *** *p* < 0.001; ** *p* < 0.01.

Figure 2 shows the mediation analysis of difficulties in emotion regulation subdimensions in the relationship between experiential avoidance and somatic symptoms, controlling for the age, income, education, and gender covariates. All reported coefficients are unstandardized (b). Experiential avoidance was significantly and positively associated with all mediators, including nonacceptance of emotional responses (b = 0.150, SE = 0.011, *p* < 0.001), difficulties engaging in goal-directed behavior (b = 0.158, SE = 0.012, *p* < 0.001), impulse control difficulties (b = 0.104, SE = 0.010, *p* < 0.001), limited access to emotion regulation strategies (b = 0.265, SE = 0.017, *p* < 0.001), and lack of emotional clarity (b = 0.074, SE = 0.006, *p* < 0.001). When all mediators and covariates were entered simultaneously to predict somatic symptoms, only difficulties in accessing emotion regulation strategies significantly predicted somatic symptoms (b = 0.317, SE = 0.105, *p* = 0.003).

The direct effect of experiential avoidance on somatic symptoms was not significant (b = 0.023, SE = 0.027, *p* = 0.401). Among the covariates, education (b = −0.954, SE = 0.359, *p* = 0.008) and gender (b = −1.534, SE = 0.525, *p* = 0.004) were significantly associated with somatic symptoms. Bootstrap analyses based on 5000 samples and 95% confidence intervals indicated that the total indirect effect of experiential avoidance on somatic symptoms was significant (b = 0.173, BootSE = 0.019, 95% CI [0.136, 0.211]).

Examination of specific indirect effects indicated that only the indirect effect through difficulties in accessing emotion regulation strategies was significant (b = 0.084, BootSE = 0.029, 95% CI [0.024, 0.141]), while the indirect effects via nonacceptance (b = 0.021, 95% CI [−0.022, 0.063]), goals (b = 0.035, 95% CI [−0.006, 0.078]), impulse control (b = 0.011, 95% CI [−0.015, 0.040]), and clarity (b = 0.023, 95% CI [−0.006, 0.053]) were not significant, as their confidence intervals included zero.

Table 3 presents specific indirect associations for each mediator. Bootstrap confidence intervals indicated that only the indirect association through limited access to emotion regulation strategies was statistically significant, whereas the indirect associations through nonacceptance, goals, impulse control difficulties, and lack of emotional clarity were not significant, as their confidence intervals included zero. The total indirect effect was statistically significant.

## 4. Discussion

The present study investigated the mediating role of emotion regulation difficulties in the relationship between experiential avoidance and somatic symptoms as well as their mutual associations. A significant association was found among the variables, in line with the existing literature. In addition, the relationship between experiential avoidance and somatic symptoms was found to be indirectly associated with difficulties in emotion regulation, suggesting that avoidant coping may be linked to difficulties in processing internal experiences, which may be reflected in somatic symptoms. Among difficulties in emotion regulation subscales, limited access to emotion regulation strategies had a significant indirect association in the relationship between experiential avoidance and somatic symptoms.

Participants reporting higher experiential avoidance scores also reported greater somatic burden. Some studies have shown an association between two variables. Mayorga et al. (2022) demonstrated that the extent of experiential avoidance and somatic complaints such as fatigue and pain were positively and significantly associated during the COVID-19 pandemic. Erbildim and Nweke (2024) analyzed the mediating role of experiential avoidance in the relationship between perceived stress and somatic symptoms. The study concluded that experiential avoidance predicted somatic symptoms, and two constructs were positively correlated. Selker et al. (2024) also investigated how acceptance of internal experiences and somatic symptoms are related. In their study, psychological flexibility, which refers to adopting an accepting attitude towards personal experiences without judgment, was relatively and significantly lower in the group with a diagnosis of somatic symptoms and related disorders, indicating a negative relationship between acceptance and persisting physical complaints. The literature and current study show that individuals with somatic burden also report higher levels of experiential avoidance. This finding can be attributed to somatic symptoms occurring and persisting due to internal experiences left dysregulated with long-term avoidance. Engaging in experiential avoidance as a coping mechanism leaves emotional burden unprocessed and is linked to physical symptoms with underlying psychological distress.

Participants who reported higher levels of experiential avoidance also had higher scores regarding difficulties in emotion regulation. Some studies have obtained similar findings regarding the association between the two variables. Yaztappeh et al. (2024) revealed that experiential avoidance and difficulties in emotion regulation are associated among individuals with stuttering. De la Cruz et al. (2013) investigated how experiential avoidance and difficulties in emotion regulation are related among individuals with hoarding disorder and concluded that the two variables are positively correlated. Shi et al. (2016) also indicated that individuals with high scores regarding emotion regulation abilities engaged in experiential avoidance less often. Experiential avoidance and difficulties in emotion regulation are maladaptive approaches for emotional coping. Both are associated with increased levels of psychological distress in the long term and development of psychopathologies. Individuals who engage in experiential avoidance are less likely to regulate their emotions with effective strategies as emotional awareness and acceptance are limited. Acceptance and awareness are considered the initial steps for modulating emotions with relevant coping approaches. This finding can also be explained by reliance on maladaptive emotion regulation strategies being linked to experiential avoidance. Individuals who struggle with emotion regulation might suppress or avoid emotional experiences to experience relief from distress.

Difficulties in emotion regulation are found to be positively and significantly related to somatic symptoms. Some studies have demonstrated the relationship between the two variables. Kang et al. (2025) illustrated that somatic symptom burden is positively related to a lack of emotional awareness. They analyzed two groups of participants with low and high levels of somatic symptoms, and for both groups, a lack of emotional awareness was found to be correlated with persisting bodily symptoms. Erkic et al. (2018) analyzed emotion regulation processes for patients with somatic symptom disorder. They demonstrated that effective emotion regulation strategies were less likely to be adopted, while most patients reported employing maladaptive approaches for emotional coping. Cognitive reappraisal, which refers to evaluating and interpreting a situation, was one of the least preferred emotion regulation approaches. The most reported strategy was expressive suppression, which involves avoiding emotional responses and is associated with somatic symptoms, inhibiting emotion processing in the long term. Garnefski et al. (2017) found that somatic burden is closely associated with preference for emotion regulation strategies. The study revealed that maladaptive attempts such as self-blame, ruminating, and catastrophizing regarding a past event are related to the severity and significance of somatic symptoms. Physiological and physical responses in the presence of an emotional or external stimulus are examined. Zhao et al. (2025) demonstrated that specific heart rate features can be identified in relation to nicotine vaping behavior, highlighting that physiological responses to an external event can be identified and measured. Low-frequency heart rate variability (LF-HRV), an index of sympathetic cardiac control, was also a correlate of emotional dysregulation according to Cattaneo et al. (2021). Overall, somatic symptoms and difficulties in emotion regulation are associated, meaning individuals with somatic burden are also expected to lack effective emotion regulation strategies or adopt ineffective ones in times of emotional distress. This can be attributed to physical manifestations of emotions when they are suppressed instead of resolved. Furthermore, chronic somatic pain, such as headaches and brain fog, might reduce one’s motivation and willingness to adopt effective emotion regulation approaches. As a result, these individuals might be discouraged from engaging in effective emotion regulation strategies that might require cognitive and physical endurance.

Difficulties in emotion regulation had a mediating role in the relationship between experiential avoidance and somatic symptoms. Avoiding internal experiences such as emotions, memories, and thoughts that are considered distressing is linked to limited cognitive and emotional capacity for emotional processing. As a result, these experiences may manifest as somatic symptoms. Emotional awareness and identification are necessary for effective application of emotion regulation strategies.

When controlling for age, gender, level of education, and income, the results indicated that limited access to emotion regulation strategies was the dimension of difficulties in emotion regulation that had a mediating role between experiential avoidance and somatic symptoms. Experiential avoidance might limit one’s motivation and willingness to apply emotion regulation strategies as this requires mental effort for awareness, understanding, and reevaluation of emotional experiences to adjust their intensity. For example, coping with depressive emotions might require both cognitive approaches such as reframing and behavioral engagement such as regular exercise. In a state of avoidance, one might lack the openness and willingness necessary for engaging in healthy, adaptive behavioral patterns. Secondly, one might perceive existing emotions that are too intense and distressing to cope with as having catastrophic consequences, such as being unable to function or experiencing unbearable shame if these emotions are seen as unacceptable or inconvenient. Thus, engaging in experiential avoidance for short-term relief can be seen as more adaptive. Finally, avoidant individuals might be less likely to acquire knowledge and experience about emotion regulation strategies as they engage in avoidant behaviors such as social withdrawal, suppression of emotions, and maladaptive activities, including excessive alcohol consumption or overworking during times of perceived stress.

Finally, education and gender were significantly associated with somatic symptoms, suggesting that sociodemographic factors may play a role in the experience of bodily symptoms. Education level negatively predicted somatic symptoms, meaning higher levels of education were associated with less somatic burden. Well-educated participants might be more open to acquiring theoretical and practical knowledge to cope with physical symptoms rather than ignoring them. They might seek medical and psychological help, diagnosis, and treatment for these symptoms, and experience relief. Female participants also reported higher scores on somatic symptoms. This can be explained by female participants having relatively more awareness regarding bodily sensations and experiences. Thus, they might be more likely to report the actual intensity of somatic symptoms.

There are some limitations of the study that can be improved in further studies to increase generalizability and reliability. Firstly, the participants lack cultural diversity. Repeating the study with participants from different cultures expected to increase the generalizability of the findings for a broader population. Secondly, the study is cross-sectional as data were collected at a single point in time. Longitudinal or prospective designs would be valuable to indicate the stability of these relationships over time. Thirdly, although several demographic variables were controlled for, other potential confounding variables such as personality traits or any medical diagnosis are not considered in the mediation analysis. Conducting a future study with a broader range of control variables would address this impact. Furthermore, the findings rely on self-report measures, which may be subject to response biases and common method variance, potentially impacting the measured associations. Employing multi-method approaches such as tasks to measure emotion regulation and assessment of bodily symptoms could provide objective indicators for somatic symptoms and emotion regulation difficulties. Finally, the results can also be interpreted considering alternative paths and explanations. Given the cross-sectional design, a causal interpretation is not possible, and the direction of the relationship cannot be established. In addition, reciprocal relationships may exist among these variables such that experiential avoidance, emotion regulation difficulties, and somatic symptoms influence each other over time. Future longitudinal or prospective studies might address the direction of the relationship.

## 5. Conclusions

In summary, experiential avoidance, difficulties in emotion regulation, and somatic symptoms were significantly interrelated to each other according to the data analysis. Difficulties in emotion regulation had an indirect effect on the relationship between experiential avoidance and somatic symptoms.

These findings provide implications for mental health professionals working with clients with co-occurring emotion regulation difficulties and somatic symptoms and who experience distress in their lives. Individuals with somatic complaints often experience anxiety about the underlying causes of these symptoms at the same time. Psychoeducation explaining the possible roles of emotion regulation difficulties and experiential avoidance might provide relief and reduce anxiety related to these symptoms. In addition, through being encouraged to adopt effective emotion regulation skills with less experiential avoidance, these clients can be provided with an alternative approach to reduce the severity of the somatic symptoms attributed to these variables. Limited access to emotion regulation strategies can be addressed to contribute to the psychological well-being of these clients. Acceptance-based approaches such as acceptance and commitment therapy, compassion-focused therapy, and mindfulness are particularly well-suited as emotion regulation approaches.

This study contributes to the existing literature by providing possible explanation regarding how affect-related processes can relate to bodily symptoms. The findings highlight that specific domain of difficulties in emotion regulation, might play a role in the somatization of emotional experiences when using avoidant approaches. This understanding of emotion regulation difficulties as a multidimensional construct is expected to provide more insights into specific pathways underlying somatic symptoms.

## Figures and Tables

**Table 1:** Descriptive statistics.

	Minimum	Maximum	Mean	Std. Deviation	Skewness	Kurtosis
Somatic symptoms	0	32	8.31	6.43	0.83	0.24
Experiential avoidance	14	81	44.71	12.96	0.09	−0.42
Difficulties in emotion regulation	15	80	36.01	15.26	0.48	−0.60
Nonacceptance	3	15	6.84	3.56	0.69	−0.53
Goal	3	15	8.60	3.63	0.026	−1.03
Impulse	2	15	5.54	2.92	1.13	0.72
Strategies	5	25	11.62	5.66	0.55	−0.76
Clarity	2	10	3.40	1.87	1.44	1.66
Age	23	80	44.47	12.93		

**Table 2:** Correlations among experiential avoidance, somatic symptoms, and difficulties in emotion regulation.

Variables	1	2	3	4	5	6	7	8
1—Somatic symptoms	1	0.42 **	0.58 **	0.50 **	0.52 **	0.43 **	0.56 **	0.37 **
2—Experiential avoidance	0.42 **	1	0.66 **	0.57 **	0.59 **	0.47 **	0.63 **	0.50 **
3—Difficulties in emotion regulation	0.58 **	0.66 **	1	0.87 **	0.87 **	0.80 **	0.94 **	0.65 **
4—Nonacceptance	0.50 **	0.57 **	0.87 **	1	0.66 **	0.60 **	0.80 **	0.52 **
5—Goal	0.52 **	0.59 **	0.87 **	0.66 **	1	0.63 **	0.81 **	0.47 **
6—Impulse	0.43 **	0.47 **	0.80 **	0.60 **	0.63 **	1	0.68 **	0.55 **
7—Strategies	0.56 **	0.63 **	0.94 **	0.80 **	0.81 **	0.68 **	1	0.51 **
8—Clarity	0.37 **	0.50 **	0.65 **	0.52 **	0.47 **	0.55 **	0.51 **	1

** *p* < 0.01.

**Table 3:** Indirect effects of experiential avoidance on somatic symptoms through difficulties in emotion regulation subdimensions controlling for age, gender, education, and perceived income.

Mediator	Indirect Effect	BootSE	95% CI
Nonacceptance	0.021	0.022	[−0.022, 0.063]
Goals	0.035	0.021	[−0.006, 0.078]
Impulse	0.011	0.014	[−0.015, 0.040]
Strategies	0.084	0.029	[0.024, 0.141]
Clarity	0.023	0.015	[−0.006, 0.053]
Total Indirect Effect	0.173	0.019	[0.136, 0.211]

## Data Availability

The data presented in this study are available from the corresponding author upon reasonable request.
